# On Some Points in the Therapy of Aconite

**Published:** 1880-06-20

**Authors:** J. W. Hickman

**Affiliations:** Delta, Pa.


### ON SOME POINTS IN THE THERAPY OF ACONITE.

BY J. W. HICKMAN, M. D., DELTA, PA.

Though an old remedy, aconite does not enjoy that degree of popularity to which it is entitled. In its sphere of influence it is one of the most valuable agents we possess. Its extended employment by homeopathists has tended largely to arouse a prejudice against it among regular practitioners; its use by those quacks, however, does not detract one jot from its real worth. In this paper we shall omit all theoretical considerations, and shall state only those facts which experience has tested, and which may hence be regarded as perfectly trustworthy.

Aconite depends for its activity upon the aconitine or aconitia which it contains; the other alkaloids which have been thus far discovered are aconella and napellina. An important point to be distinctly understood at the outset is that aconite is a drug possessing, upon the basis of dosage, a two-fold and opposite action; that is, the effect of a small dose is diametrically opposite to the effect induced by a large dose. This may be best seen in its influence upon the circulation; a small dose as a drop or two will slow the heart's action, while a large one will hasten its activity. Any one may test the truth of this statement for himself. Without an appreciation of this fact it is utterly impossible to derive satisfactory results from the administration of aconite. In small doses aconite will be found to control the heart, both in point of frequency and power of contraction. It also lessens arterial tension, and increases elimination by the skin; and, according to some authorities, it increases the functional activity of the kidneys. Most observers will, I think, agree that its diuretic tendency is not constant, however. To possess an accurate knowledge of its therapeutics, then, it becomes us to seek after the conditions upon which this variance depends. G. Hunter McKenzie tells us (Practitioner, Jan., 1880), as his conclusion from a series of careful observations, that in cases marked by an absence of cardiac and renal diseases, and in certain febrile conditions, aconite has a tendency to increase the urinary secretion. Every practitioner can, in cases requiring the use of aconite, test for himself the accuracy of these conclusions.

Aconite will also slow the respiratory movements, and will lessen abnormal heat. The heart, however, seems to receive the brunt of its action; for, in case of poisoning, the cardiac movements cease before those of the respiration. The systemic effects of the drug are manifested in about thirty minutes, and last for about three hours; hence the dose should be repeated at about this interval. Of course, aconite is contraindicated in all conditions of adynamia. It should be remembered, too, that aconite will increase an existing irritability of the gastric mucous membrane, thus impairing the appetite, hindering digestion and causing diarrhea. In the normal state of the mucous membrane, however, it will increase the gastric secretions and accelerate the peristaltic movements.

In addition to the measures usually instituted in cases of poisoning by aconite, digitalis should be recognized as a valuable antagonistic to the depressing influence of the drug in question. It was first recommended by Fothergill. In a recent clinical lecture Professor DaCosta recommends the hypodermic injection of two drops of the fluid extract of digitalis in water, in any case of heart-failure from whatever cause; this is at once an efficient, prompt and convenient method of administration, and richly deserves to be held in memory, not only in aconite poisoning, but in like conditions generally. In such of the acute inflammatory and febrile conditions as are especially marked by sthenic reaction (high temperature, increased arterial tension and rapidity of pulse), aconite is a highly valuable agent. The continued fevers form an exception to this statement, save where great hyperpyrexia obtains.

This drug interferes in no way with other measures indicated in these conditions; it does faithfully its own special work, and leaves the attendant free to meet other indications as they present themselves. Aconite takes no second rank in the treatment of those *acute inflammations of the respiratory organs* which are at all extensive. Than this drug there is no more reliable in acute *pneumonia* and pleurisy.

But a few days ago I prescribed two drops of the tincture of the root every three hours in a case of pneumonia at the base of the right lung. There was, when first seen, on the fifth day after the invasion, delirium with a temperature in the axilla of 103.4°, and the pulse, full and bounding, was driving away at a rate of 132 per minute. At nearly the same time on the next day the thermometer registered but 100.1°, and the circulation was fully under control at 85 pulsations per minute. The aconite was the only agent given internally; of course poultices were applied externally.

Nor is this an isolated case; I have seen the same good result over and over again. As a rule, aconite is the only remedy necesssary up to the period of crisis. It will be found useful at any stage, however, for it not only moderates the activity of the symptoms, but assists in the removal of inflammatory products. Pleurisy, too, up to the stage of effusion, is best treated by small doses of aconite. If there be severe pain, it will be well to combine it with some preparations of opium.

℞ Tinc. aconiti rad.................................................... ʒ ij.

Tinc. opii deodorat.................................................... ʒ vi.

Dose, eight drops in water every two or three hours, or at first oftener. With poultices externally no treatment is more efficient.

The hot stage of intermittent and remittent fevers may be greatly modified in intensity by drop-doses of aconite every half-hour or hour. It may be conveniently given in some fever mixture as the effervescing draught or neutral mixture. Aconite is almost indispensable in the treatment of certain stages in scarlet fever—the eruptive stage and the stage of desquamation. It not only lowers the temperature, but it increases elimination by the skin and kidneys, and tends to check the nasal, faucial and aural inflammations which constitute such troublesome complications and such terrible sequelæ. In measles where the temperature runs high, or where acute pneumonia or bronchitis threatens, aconite is by all odds our best remedy; it will control the tendencies of the disease as no other agent can.

Peritonitis is best treated by a combination of aconite and opium, similar to that recommended above for pleurisy; larger doses of opium will be required, however. Aconite is a valuable remedy in cerebral and cerebro-spinal meningitis, and also in the active form of acute cerebral congestions, occurring within the cranial cavity. The very best remedy we can employ in cardiac hypertrophy is aconite, in one or two-drop doses, three times a day, for a week or two; it is then well to drop to twice a day, of course watching carefully the effect of a protracted use. As a rule the drug must be continued for six or nine months. In addition to this, the exciting cause must be removed, and the patient lie down a few hours each day; thus every indication of the case is at once met, and more than this plan of treatment nothing can be done.

We are told by Ringer and by Phillips that suppression of the catamenial flow, caused by cold, can be relieved by drop doses of aconite, repeated every half-hour or hour. In intense traumatic inflammations it is well to combine aconite with a small quantity of sulphate of morphia.

Much has lately been written as to the efficacy of aconitia in neuralgia. I cannot speak from experience with the remedy, but it is recommended by authorities whose capability or veracity cannot for one minute be called in question. Dr. Robert F. Weir, Surgeon to the New York and Roosevelt Hospitals, reports a case of eighteen years standing which he cured by this agent. The affection was, in the main, confined to the distribution infra-orbital nerve of the left side of the face, with the paroxysm recurring nearly every minute. Sleep had been obtained by chloral and opium. At first 1–140 of Duquesnel's preparation was exhibited three times per day. In two days it was increased to 1-96 grs. *per die*. After another two days four doses per day; no physiological effect, although the pain was relieved. After eleven days from the first dose, seven doses of 1–96 grs. were given per day; no physiological effect save a slight chilliness. The pain soon left him entirely. Dr. E. C. Sequin prescribes it thus :

℞ Duquesnel's aconitia ........................................ gr. 1-12 to ⅛.

Alcohol..................................................................

Glycerin ........................................................aa ʒj.

Peppermint water.......................................ad. f. ℥ ij. M

Dose, one teaspoonful three times per day. The dose is to be steadily but carefully increased. He sometimes finds it to succeed perfectly, and again it proves absolutely worthless. We can only say that, in the absence of other effective means, this agent merits a persistent trial. As a local application in painful conditions, Bartholow recommends the following :

℞ Tinc. aconiti rad.................................................

Chloroformi..................................................aa ℥ ss.

Lin. saponis............................................................ ℥j. M

Dose, moisten a piece of flannel and apply to painful point. He says it is much more efficient than the officinal aconite liniment.—*Country Practitioner*.

---

Faraday says, in a lecture, that Society, speaking generally, is not only ignorant as respects education of the judgment, but is also ignorant of its ignorance; correct judgment with regard to surrounding objects, events, and consequences becomes possible only through knowledge of the way in which surrounding phenomena depend on each other.

